# Function and dysfunction of the dystonia network: an exploration of neural circuits that underlie the acquired and isolated dystonias

**DOI:** 10.3389/dyst.2023.11805

**Published:** 2023-12-13

**Authors:** Jason S. Gill, Megan X. Nguyen, Mariam Hull, Meike E. van der Heijden, Ken Nguyen, Sruthi P. Thomas, Roy V. Sillitoe

**Affiliations:** 1 Division of Neurology and Developmental Neuroscience, Department of Pediatrics, Baylor College of Medicine, Houston, TX, United States; 2 Jan and Dan Duncan Neurological Research Institute at Texas Children’s Hospital, Houston, TX, United States; 3 Department of Pathology and Immunology, Baylor College of Medicine, Houston, TX, United State; 4 H. Ben Taub Department of Physical Medicine and Rehabilitation, Baylor College of Medicine, Houston, TX, United States; 5 Department of Neurosurgery, Baylor College of Medicine, Houston, TX, United States; 6 Department of Neuroscience, Baylor College of Medicine, Houston, TX, United States; 7 Development, Disease Models and Therapeutics Graduate Program, Baylor College of Medicine, Houston, TX, United States

**Keywords:** dystonia, cerebellum, dyskinetic cerebral palsy, dystonia network, network disorder

## Abstract

Dystonia is a highly prevalent movement disorder that can manifest at any time across the lifespan. An increasing number of investigations have tied this disorder to dysfunction of a broad “dystonia network” encompassing the cerebellum, thalamus, basal ganglia, and cortex. However, pinpointing how dysfunction of the various anatomic components of the network produces the wide variety of dystonia presentations across etiologies remains a difficult problem. In this review, a discussion of functional network findings in non-mendelian etiologies of dystonia is undertaken. Initially acquired etiologies of dystonia and how lesion location leads to alterations in network function are explored, first through an examination of cerebral palsy, in which early brain injury may lead to dystonic/dyskinetic forms of the movement disorder. The discussion of acquired etiologies then continues with an evaluation of the literature covering dystonia resulting from focal lesions followed by the isolated focal dystonias, both idiopathic and task dependent. Next, how the dystonia network responds to therapeutic interventions, from the “geste antagoniste” or “sensory trick” to botulinum toxin and deep brain stimulation, is covered with an eye towards finding similarities in network responses with effective treatment. Finally, an examination of how focal network disruptions in mouse models has informed our understanding of the circuits involved in dystonia is provided. Together, this article aims to offer a synthesis of the literature examining dystonia from the perspective of brain networks and it provides grounding for the perspective of dystonia as disorder of network function.

## Introduction

Dystonia refers to the involuntary intermittent or sustained contraction of muscles resulting in abnormal, repetitive movements (such as tremor) or postures. Dystonia occurs in isolated muscle groups (focal), contiguous muscle groups (segmental), or in groups distributed across the body (generalized) (See videos in Table 1 for examples) [1, 2]. Dystonia is estimated to be the 2nd or 3rd most common movement disorder, though the true prevalence is likely underestimated due to the paucity of studies on prevalence and the exclusion of cases in which those with, for instance, mild focal or task-specific dystonia may not seek medical attention [3].

Dystonia classification schemes have varied over time [3, 4]. The usage of “primary” and “secondary” dystonia, which have been used in varying ways to describe dystonia arising from hereditary, neurodegenerative, acquired, and idiopathic causes has recently been revisited [1, 4]. Confusing the older classification scheme is its inability to distinguish the phenomenology from the etiology of the dystonia, two important aspects of the disease that may at times be at odds with one another [1]. In an attempt to account for the dichotomy arising from etiologic and phenomenological considerations in dystonia, a recent classification scheme based on expert consensus aligned the wide variety of dystonias along two axes [1]: Axis 1 classifies the clinical characteristics of the disease presentation and Axis 2 classifies known etiology. Importantly, this scheme was proposed with the understanding that the dystonia afflicting a given individual could evolve along each of these axes independently and/or in parallel as the disease evolves and/or more information about the patient’s condition is obtained.

The treatment for a given dystonia can be thought of as an independent classification consideration that can relate to either axis, being more closely related to etiology in the case of deep brain stimulation (DBS) and phenomenology in the case of botulinum neurotoxin (BoNT). In general, dystonias that were formerly classified as primary dystonias, including genetic and focal/segmental idiopathic dystonias, are more amenable to treatment [5] while dystonia due to lesions or acquired injury are often more difficult to treat. The former group (previously “primary”) includes the dopa-responsive dystonias and idiopathic dystonias that are often responsive to pallidal deep brain stimulation [6, 7]. Treatment of dystonia is often multifactorial, first targeting reversible causes such as discontinuation of the putative offending agents, as well as consideration of disease-specific therapies. When there are no disease specific treatments available, supportive measures are used that include oral medications, botulinum neurotoxin injections, or surgical interventions such as intrathecal baclofen pump or deep brain stimulation [2, 8–13]. This review will discuss the increasingly large body of literature that exists regarding the interrogation of 1) the network dysfunction underlying dystonia and 2) how therapeutic modalities act upon those dysfunctional networks.

The genetic dystonias have been thoroughly reviewed due to their prevalence and the relative ease with which diseases with mendelian inheritance can be interrogated in the genomics era, both through clinical research and with model organisms [2, 14]. The present review will focus on acquired and idiopathic dystonia from the perspective of brain networks with the hopes of highlighting possible commonalities between what may seem to be disparate forms of the disease along both dystonia axes. This approach takes advantage of the network specificity derived from lesion mapping—how damage to the brain is associated with network changes—and functional brain imaging—how brain networks are operating in real time—to examine how recent advances in research have come to characterize this movement disorder as having a basis in dysfunction of a “dystonia network” [15–17]. The primary nodes of the dystonia network include the cerebellum, the thalamus, the basal ganglia, and sensorimotor cortical regions [18–20]. However, higher order associative cortical regions (as will be described below) as well as deep structures that link these regions, including the midbrain, pons, and brainstem, likely have an ancillary role in dystonia pathogenesis [15, 21, 22].

To understand how dystonia network dysfunction manifests, first we will evaluate the acquired dystonias in comparison to network function in idiopathic dystonias. Next, we will evaluate how interventions that alleviate dystonia work by correcting or modulating the dysfunctional networks. Finally, we will look at mouse models both of lesion associated dystonia and of dystonia elicited by direct network manipulation. These perspectives on dystonia etiology complement one another and we hope will offer a fresh synthesis working towards the understanding of how network dysfunction contributes to dystonia and what that tells us about broader disease etiology. We hope that this perspective will help inform therapeutic interventions in the dystonias that have remained intractable to therapy through an emphasis on understanding the neural substrates of the disease.

## Acquired, lesion-associated, and isolated idiopathic focal/segmental dystonia

Acquired forms of dystonia may be placed into two broad categories, those with a structural lesion within the central nervous system (CNS) and those with no clear structural abnormalities. Dystonia resulting from structural abnormalities can further be divided into those that occur early in life and are the result of more widespread CNS damage (seen in dyskinetic/dystonic cerebral palsy; Supplementary Video S1) and those that are the result of later, focal CNS lesions (i.e., ischemic/hemorrhagic stroke; Supplementary Video S2). An interesting counterpart to the focal lesion associated dystonias are the drug induced dystonias (tardive dystonia and acute dystonic reactions) and isolated idiopathic focal/segmental dystonias, which may be thought of as focal functional network disruptions, and which will be covered in the final portion of this section. Finally, outside the scope of this discussion, but interesting from the perspective of network dysfunction is functional/psychogenic dystonia of which the etiology remains more enigmatic (Supplementary Video S3); whether functional/psychogenic dystonias share the network abnormalities of idiopathic dystonia or merely converge on Axis 1 will be an interesting problem to untangle moving forward.

### Dystonic cerebral palsy

Cerebral Palsy (CP) is the most common etiology of severe motor dysfunction in childhood [23]. While CP has traditionally been associated with spasticity, there is an increasing awareness that individuals with CP frequently experience dystonia, even to the point that is may be present in the majority of these patients [24, 25]. In addition to the hypertonia and movement disorders seen in CP, a wide variety of co-morbid neurodevelopmental disabilities may also be present, including epilepsy, autism spectrum disorders, and intellectual disability, implicating pathology across distributed brain networks [26–30]. Furthermore, CP is not an etiologic diagnosis but one made based on the timing of the presumed central nervous system insult (pre-, peri-, neo-natal), the course of the disease (stable rather than progressive), and clinical presentation [31]. CP thus offers a unique opportunity to evaluate the intersection of lesion location and clinical outcome. In particular, dystonic presentations of CP present an excellent opportunity to understand the genesis of acquired forms of dystonia and their associated underpinnings in network dysfunction because there is an early, acquired insult and subsequent dystonia [32].

**FIGURE 1:**
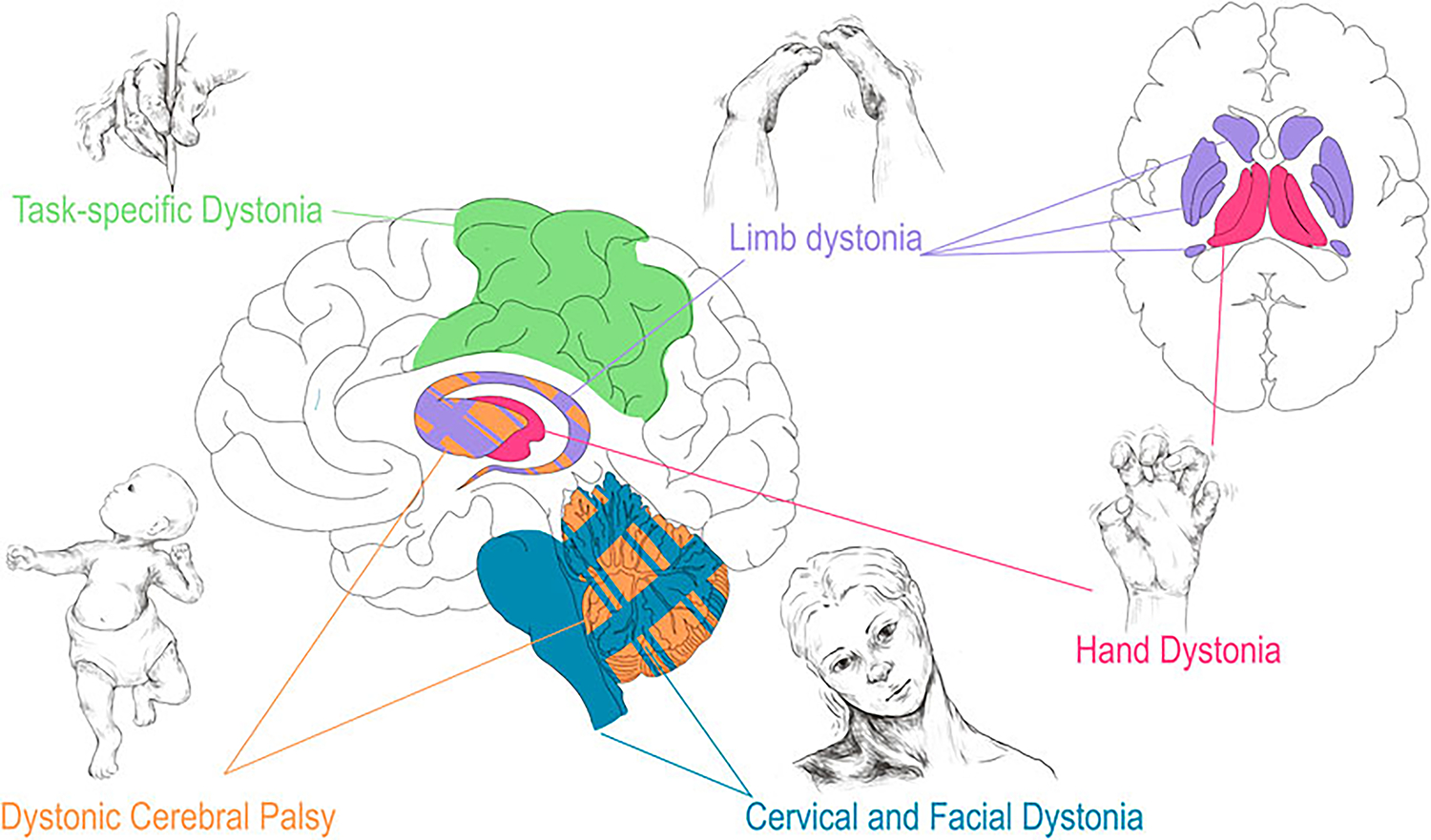
Correlation of dystonia presentation with corresponding regional brain disturbance. Dystonia associated with CP (orange) may involve injury to all highlighted structures especially basal ganglia and cerebellum. As described in Corp et al. the presentation of focal, lesion associated dystonia has correlates with area of brain injured: limb dystonia often localizes to lesions in the basal ganglia (purple), hand dystonia to lesions of the thalamus (red), and cervical dystonia/blepharospasm to lesions of the cerebellum and brainstem (teal) [41]. Task specific dystonia (green) shows predominantly neocortical network abnormalities upon a shared base of subcortical dysfunction when compared to non-task idiopathic dystonia [42]. Drawings are original works that were made in Procreate Version 5.3.5 and the figure was compiled in Adobe Illustrator Version 24.0.1.

Numerous structural brain lesions are associated with CP. Periventricular leukomalacia (PVL), which is associated with premature birth and intraventricular hemorrhage (hemorrhage of the germinal matrix along the walls of the lateral ventricles), is the radiographic abnormality most commonly seen in CP. While PVL refers specifically to the direct damage to the white matter tracts along the lateral ventricles, larger intraventricular hemorrhages [33] risk the disruption of subcortical brain structures including the thalamus, basal ganglia, and cerebellum [34–36]. Furthermore, hypoxic ischemic encephalopathy (HIE), unconjugated hyperbilirubinemia due to kernicterus, and disruption of cerebellar development associated with birth early in the third trimester are additional acquired perinatal insults associated with CP [37–40]. As mentioned, the cerebellum, thalamus, basal ganglia, and sensorimotor cortex encompass the key nodes of the dystonia network [15–17]. As will be seen throughout this review, damage and dysfunction to various parts of this network underlies many forms of dystonia. Dystonic/dyskinetic CP is unique among the acquired dystonias in that it, as described above, often encompasses damage to various structures simultaneously (Figure 1).

Several reports have investigated how subcortical regions are affected in patients with dystonic/dyskinetic CP. To start, a population-based study in CP patients investigated imaging finding in patients with all CP subtypes (*n* = 213) as well as those with dystonic/dyskinetic CP (*n* = 15) in particular [43]. While the majority of patients (87%) had cerebral abnormalities on imaging (including PVL, cortical gray matter injury, evidence of stroke, and cerebral malformations), dystonic/dyskinetic CP patients had either non-specific changes (7/15; delayed myelination or volume loss) or normal imaging (4/15) findings [43]. Along these lines, a more recent study looked at imaging in patients with acute HIE at day 4–5 of life; they found that evidence of cytotoxic edema (ADC map on magnetic resonance imaging (MRI)) to the thalamus and striatum was highly predictive of subsequent dystonia [44]. Notably, diffuse low grade cytotoxic edema, in contrast to the dense cytotoxic edema associated with ischemic stroke, may lead to more subtle volume loss and perhaps explains the findings noted above associating mild imaging abnormalities with dystonic CP. Consistent with the lack of gross cerebral abnormalities in patients with purely dystonic CP [43], a more recent analysis found that patients with dystonic/dyskinetic CP (*n* = 39) were likely to have MRI abnormalities of low graded severity (sqMRI score < 5.5/40), but with multiple brain regions affected (most often a combination of cortical and thalamo-striatal findings) [45]. Furthermore, patients with dystonic/dyskinetic CP were likely to have severe motor disability despite relatively less severe imaging abnormalities with the majority of dystonic CP patients scoring a IV or V on the gross motor function classification scale [46], where higher numbers (scale I–V) indicate more severe disability. A supposition reconciling the disparity between the findings on imaging and severity of motor disability may be that dystonic CP results from a functional impairment of the dystonia network that is compounded due to the involvement of multiple nodes in the network. Also to consider is that the dystonic/dyskinetic CP patients with nonspecific/normal imaging findings may also have undiagnosed genetic or metabolic conditions, which could contribute to more global network dysfunction [47]. In fact, an important caveat to keep in mind in the case of nearly all imaging studies in acquired and idiopathic dystonia is that underlying, unknown monogenic or non-mendelian inherited pre-dispositions to developing dystonia are an active area of study that may modify the thinking on these dystonias as our knowledge expands.

Indeed, more recent studies using higher level structural and functional imaging modalities have found that there is evidence of broad network abnormalities in patients with dystonic/dyskinetic CP. Ballester-Plané et al. found widespread alterations in the cortico-cortico, subcortical-cortico, and intra-subcortical white matter networks using tractography [48]. Consistent with the previously described results, they found that these network alterations were present even in patients whose conventional MRIs failed to show abnormalities [48]. Interestingly, they found there were certain network nodes that showed increased nodal connectivity (right pallidum with supramarginal gyrus) indicating that abnormal network response to injury/dysfunction may also be a component of dystonic/dyskinetic CP [48]. The authors did note a caveat that they did not analyze cerebellar tractography, which would certainly be interesting to examine. Another study from Wu et al. built upon these findings, noting that in CP patients with normal MRIs there were functional impairments in glucose metabolism on FDG PET in several cortical regions, cerebellum, and the “central region” [49].

Qin et al. next undertook two studies using functional MRI (fMRI), which is an imaging modality that identifies increased regional brain activity using blood oxygen level dependent (BOLD) signaling during periods of directed activity (task dependent) or at rest (resting state) [50–53]. The group evaluated resting state brain network abnormalities in patients with both spastic and dystonic/dyskinetic CP in comparison to controls and to dystonic/dyskinetic CP alone [52, 53]. In the comparison study, they found that both types of CP showed abnormalities in the cerebellum and several cortical networks while individuals with dystonic/dyskinetic CP had unique cerebello-cortical functional disconnections [52]. Building upon this study, the group subsequently noted that there was altered interhemispheric connectivity in individuals with dystonic CP [53].

Finally, it must be noted that many individuals previously diagnosed with idiopathic dystonic or dyskinetic CP have been increasingly found to have underlying genetic abnormalities and dystonias, rather than acquired brain insults, which bears further targeted investigation [54, 55]. Nonetheless, susceptibility of the cortical and subcortical dystonia network to clinical entities such as PVL, germinal matrix hemorrhage, kernicterus, HIE, and direct or indirect aberrant cerebellar development predisposes individuals with perinatal brain injury to dystonia and dystonic CP. Moving forward, it will be important to continue to understand how these structural insults lead to the functional network abnormalities that underlie the resultant dystonia in order to devise more targeted, efficacious interventions.

### Lesion-associated dystonia

Dystonia as a distinct movement disorder was first described in the western medical literature by Marcus Walder Schwalbe in 1908, despite many previous descriptions of conditions that doubtless would be considered dystonia today [56]. Despite the occasional subsequent reports linking dystonia to brain lesions, debate persisted as to whether dystonia represented a psychiatric disorder or had its basis in organic brain pathology [56]. It was not until 1984 that Charles Marsden, with the support of the Dystonia Medical Research Foundation, proposed a more formalized definition of dystonia, which mirrors the one now in use. Subsequently, Marsden’s group and the group of Joseph Jankovic published work that linked the appearance of dystonia with various lesions of the central nervous system [57, 58]. From that time on, and especially with the advent of increasingly sophisticated brain imaging modalities, the understanding of how dystonia arises from lesions of the CNS has expanded enormously.

Recently, meta-analyses of lesion based dystonia studies have evaluated the link between structural lesions and related functional outcomes [41]. Confirming and building upon previous theories of lesion based dystonia, Corp et al. surveyed 359 individual cases and found that the vast majority of the lesions were in subcortical structures (approx. 93%) [41]. The basal ganglia and thalamus were the most common subcortical regions involved in dystonia, which coincides with the previously described dystonia network [41, 59]. Furthermore, injury to the basal ganglia and cerebellum were found to predispose patients to dystonia, rather than non-dystonia movement disorders, compared to lesions in other brain regions [41]. Intriguingly, Corp et al. found a correlation of lesion location to dystonia location with basal ganglia lesions more often leading to limb dystonia, thalamic lesions more often leading to hand dystonia, and cerebellar and brainstem lesions more often leading to cervical and facial dystonia (blepharospasm) (Figure 1) [41]. Future work corroborating these findings will be important to further interrogate and validate these anatomical links. Furthermore, how focal and segmental dystonias arising from lesions relate to generalized dystonia associated with hereditary and heredodegenerative conditions remains to be explored.

Further questions arise regarding how lesion related dystonia informs our understanding of functional networks and idiopathic acquired dystonias. Previous work used lesion based network mapping [60] to define the networks associated with cervical dystonia [15]. This work showed that lesions producing cervical dystonia displayed increased connectivity to the cerebellum and decreased connectivity to somatosensory cortex even given lesions located in widespread brain regions (cerebellum, basal ganglia, brainstem). This finding mirrors the “disconnection” noted by the functional and structural brain studies described in dystonic CP [52, 53]. Further exploration of lesion-based dystonia may help to uncover the interplay between networks and the nodes that are susceptible and sufficient to produce dystonia. As the focus of network analysis is expanded to broader dystonia phenotypes, the picture of functional networks involved in dystonia, and the direction (increased vs. decreased activity of a given brain region) of the functional disruption, becomes more complicated though increasingly convergent on the described dystonia network [17, 61].

### Idiopathic isolated focal dystonia

We next focus on focal dystonia without an identified genetic or structural lesion, or idiopathic isolated focal dystonia. Isolated focal dystonias are generally categorized as idiopathic dystonia (i.e., blepharospasm or some cervical dystonias) or task specific focal dystonia (i.e., writer’s cramp, laryngeal dystonia). Given the possible association with a task and the absence of anatomic disease pathology it is understandable why this class of dystonias were previously considered to be a psychiatric disorder [56]. However, with the advent of advanced MRI techniques, the network abnormalities underlying isolated dystonia have increasingly come to light [62]. The most extensively studied isolated dystonias are blepharospasm (which involves involuntary contractions of muscles of the eyes and face) and cervical dystonia (involving involuntary contractions of axial muscles of the upper back and neck) [1]. fMRI is a key tool in the investigation of these dystonias. fMRI can be used to determine functional connectivity (FC; fcMRI) by measuring how activity in a given brain region changes in relation to a defined anatomical site (region of interest (ROI) or seed) [50, 51, 63]. Initial studies examining functional connectivity in cervical dystonia implicated abnormal connectivity in the cerebello-striato-cortical network in dystonia in both the resting state as well as in task dependent fMRI [64–66]. Interestingly, several studies have shown alterations in connectivity between the cerebellum and the somatosensory cortex [66, 67], which was also seen in previous analyses of lesion based dystonia [15] and dystonic cerebral palsy [52]. Together, these findings point to the interesting conclusion that abnormal and anti-correlated sensorimotor activity in cortical and cerebellar networks is a key feature of dystonia [68, 69]. Whether this “acquired” network dysfunction in idiopathic dystonia directly parallels the pathologic network function caused by brain insult in acquired dystonia will be an interesting area of investigation moving forward. The task specific dystonias, which will be discussed next, perhaps offer an interesting perspective on this, with a higher degree of cortical abnormalities in non-somatosensory cortical regions.

A second important class of focal isolated dystonia is task specific focal dystonia and peripherally induced dystonia, of which laryngeal dystonia (previously called spasmodic dysphonia) and writer’s cramp or musician’s dystonia are the most closely studied [42]. These dystonias have the interesting feature of being associated with environmental factors or “overuse,” namely, professional use of voice in laryngeal dystonia or extensive practiced fine motor control in writer’s cramp or musician’s dystonia [70–74]. In addition, frequent case reports arise in the literature of various dystonias associated with other repetitive focused tasks including, briefly, laryngeal dystonia in a telemarketer and focal appendicular dystonias in a typist, a billiards player, a blacksmith, during braking while driving, and in a runner [75–81]. Despite the association of this class of dystonias with environmental factors, there is evidence of underlying genetic associations including both predisposing family history and the identification of genetic mutations [82, 83]. Regarding the latter, many genes that are involved in generalized or segmental dystonias may present as task specific focal dystonia [82], which does pose the question of how environment and genotype interact to affect the penetrance of dystonia and whether the genetic dystonias predispose the dystonia network to failure dependent on other coinciding factors rather than directly leading to their degeneration or dysfunction.

Given the nature of focal isolated dystonia, especially the “overuse” in task specific focal dystonia, there has been a great deal of work exploring the pathophysiology of these dystonias using fcMRI [62]. In direct comparisons, evaluation of functional networks in task specific and non-task specific isolated dystonia showed shared disruption of the subcortical dystonia networks involving the basal ganglia and the cerebellum [42]. This study by Battistella et al. further suggests that the network disruption of these two brain regions, the output of which converge on cortical areas important for the pathogenesis of dystonia, forms a “common base for propagation of larger scale network abnormalities” [42]. However, the direct comparison further revealed important distinctions between task and non-task focal dystonia, indicating divergence in pathogenesis from the shared base of subcortical network dysfunction. In particular, the authors found that task specific dystonia had broad cortical network dysfunction, which encompassed primary motor, somatosensory, and inferior parietal cortices, compared to more isolated dysfunction in the somatosensory cortex in non-task dystonia (Figure 1) [42]. The authors of the study posit that the pathology of task based focal dystonia involves the broader interaction of functional cortical domains, which is consistent with the task dependence of the acquisition of this dystonia phenotype [42]. Subsequent studies from this group further delineated differences between task specificity in different focal dystonias. Laryngeal dystonia showed involvement of some unique cortical areas (parietal cortex, inferior frontal gyrus, anterior insula) compared to musician’s dystonia (primary and secondary sensorimotor cortex and middle frontal gyrus), and shared involvement in premotor and certain parietal cortical domains [84]. Together these studies offer a conceptual leap: perhaps the lesion in task dependent focal dystonia is the aberrant cortical connectivity driven by the prolonged and concerted effort to perform the given task, which itself would require concurrent, concerted integration of striatal and cerebellar function.

Furthermore, changes in white matter integrity using diffusion weighted imaging (DWI; diffusion tensor imaging (DTI)) sequences on MRI were evaluated to delve deeper into the structural basis of the identified functional network alterations [21]. Here, it was noted that the white matter changes involved primarily basal ganglia and cortical areas, including involvement of altered “neural hub” localization in the frontal cortices, and disparate involvement of premotor and occipital cortices in laryngeal dystonia and writer’s cramp, respectively [21]. Interestingly this mirrors the findings mentioned earlier in dystonic CP [48]. The absence of structural white matter changes in the primary motor cortex and cerebellum [21], both of which are implicated in the dystonia network, raises an interesting question: are the task specific focal dystonias distinct in their underlying network pathophysiology, or do the cerebellum and motor cortex play a role in the pathogenesis of task specific dystonia that would not be reflected in DTI? For example, as DTI modalities reflect white matter changes would evidence of gray matter alterations be seen in these patients perhaps reflecting a computational role for the cerebellum and role in connectivity/plasticity for the regions implicated above? Indeed, previous studies demonstrated alterations in gray matter in the thalamus, primary sensorimotor cortex, and cerebellum in 30 patients with writer’s cramp compared to control patients [85]. Together these studies suggest the possibility of fundamentally different roles for the regions of the dystonia network that show changes in white matter compared to those that show changes in gray matter, roles that are intertwined but distinct, and apparent only with unique analytic methodologies.

## Clues to dystonia pathophysiology derived from therapeutic intervention

Recent work in understanding the network effects of therapeutic interventions in dystonia have furthered our understanding of dystonia pathogenesis. In contrast to the genetic, “primary” dystonias that often respond well to targeted pharmacology (e.g., dopa responsive dystonias) or deep brain stimulation (DBS) targeting the basal ganglia, acquired dystonias are often difficult to treat. However, investigations into how brain networks change after sensory tricks or effective treatments (botulinum neurotoxin injections, and conventional/unconventional sites of DBS) have contributed to our understanding of the pathophysiology and etiology of acquired and idiopathic dystonias. In this section, we will explore the literature investigating how therapeutic interventions inform our understanding of the underlying dystonia networks.

### Sensory trick, or geste antagoniste

The oldest non-pharmacologic intervention in focal dystonia is the “geste antagoniste” or “sensory trick,” which was first published in the western medical literature in 1893 [86]. This trick involves a motion or touch that temporarily corrects the dystonia, for instance touching the affected side of the face to resolve an episode of blepharospasm. In what seems to be a theme, the efficacy of the sensory trick was taken to be evidence for the psychiatric nature of dystonia [86]. It was not until almost 100 years later that dystonia as a disorder of sensory motor integration became the predominant viewpoint. The view of dystonia as a sensory motor disorder, rather than a functional psychiatric disorder, has held up under rigorous investigation, but how tightly dystonia straddles the line between an organic disorder of brain function and a functional psychogenic disorder is perhaps best demonstrated by a comment from Corp et al.: “The notion that cervical dystonia may be a form of sensory or proprioceptive hallucination is highly speculative, but a testable hypothesis motivated by the present findings” [15]. The efficacy of sensory tricks in primary dystonia is a key component of the current understanding that dystonia is in part a sensory disorder [87, 88]. Furthermore, dystonia is often preceded by sensory symptoms such as discomfort or pain weeks to months before the dystonia develops [89]. A study from Kägi et al. confirmed the association between sensory tricks, sensory integration, and dystonia by showing that shorter duration of dystonia and higher efficacy of sensory trick were correlated with better sensory discrimination. In contrast, failure to respond to sensory tricks was associated with longer disease duration and poor sensory discrimination. The correlation between disease duration and sensory discrimination directly imputes sensation in the disease and offers the added insight of a progressive sensory dysfunction in disease presentation [90]. Furthermore, electromyography has been used to evaluate sensory trick efficacy and has furthered our understanding of how sensory changes affect the objective findings in dystonia. In particular, Deuschl et al. used electromyography to confirm cessation of dystonia in torticollis (cervical dystonia) with the touching of the hand to the chin [91].

**FIGURE 2:**
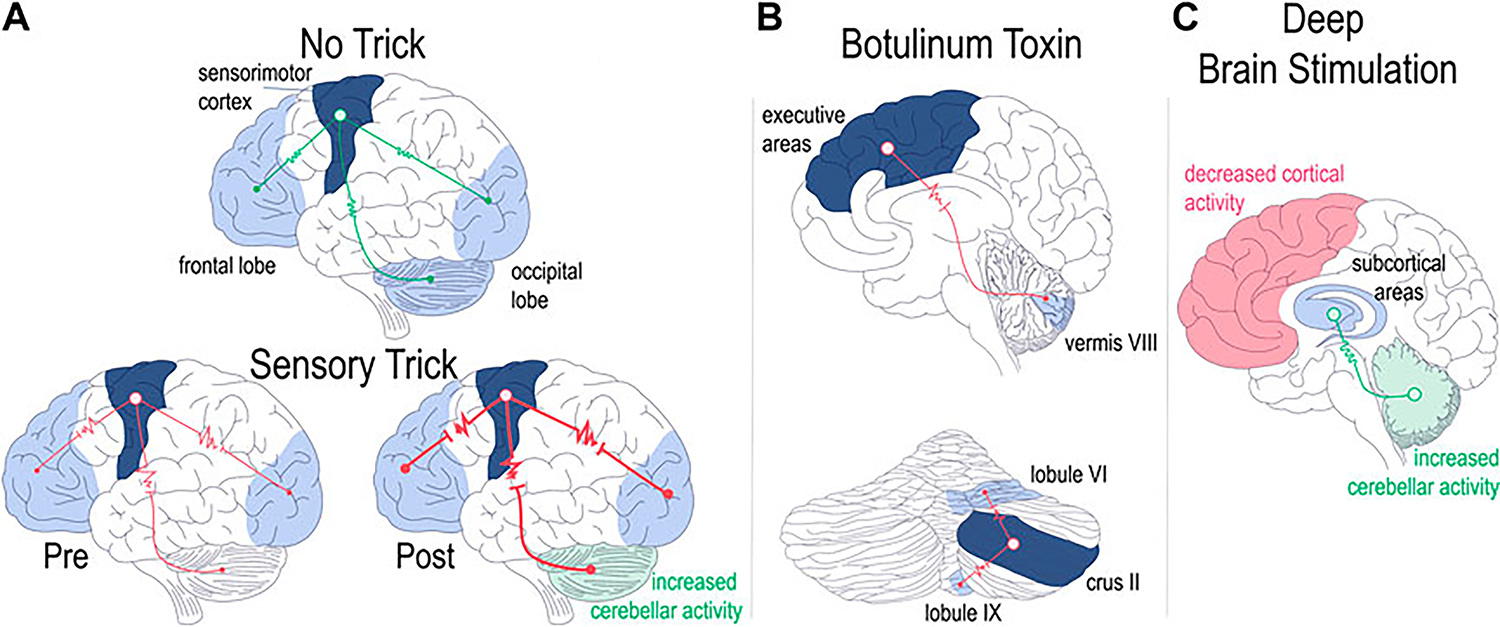
Regional brain network abnormalities associated with therapeutic interventions. Patients with dystonia non-responsive [**(A)**, upper panel] or responsive [**(A)**, lower panel] to sensory tricks showed differences in regional functional connectivity **(A)**. Patients non-responsive to sensory tricks showed increased connectivity across cerebellar and cortical networks [**(A)**, top]. Patients with dystonia responsive to sensory tricks had comparatively lower regional brain connectivity which was then further decreased with trick performance [**(A)**, bottom left vs. right]. Trick performance also led to increased cerebellar activity [**(A)** bottom right] [93]. Functional connectivity with botulinum toxin therapy **(B)**. Successful treatment of cervical dystonia with BoNT led to decreased functional connectivity between cortex and cerebellum [**(B)**, top] as well as decreased intrinsic cerebellar functional connectivity [**(B)**, bottom] [94, 95]. Optimal DBS settings lead to alterations in network functional connectivity **(C)**. Cortical areas show decreased activity while the cerebellum shows increased activity with optimal DBS settings turned on as compared to when DBS is off [96]. Optimal DBS setting also show increased connectivity of subcortical networks [97]. Drawings are original works that were made in Procreate Version 5.3.5 and the figure was compiled in Adobe Illustrator Version 24.0.1.

Extending these findings, Wissel et al. later confirmed the finding of electromyographic improvement of cervical dystonia with the sensory trick [92]. They also made the interesting observation that in just over half of the patients that they studied, electromyographic improvement *preceded* the tactile stimulation, implicating not only sensory networks in dystonia, but perhaps higher order cortical areas as well [92]. The involvement of higher order cortical areas was also alluded to by the studies from the Simonyan group detailed in the section on task specific focal dystonia [21, 42, 70]. More recently, fMRI studies confirmed the suspicion of higher order cortical involvement in sensory tricks by showing that patients with cervical dystonia have altered functional connectivity in sensorimotor, visual, and executive cortical domains [93]. Intriguingly, the authors found differences in functional connectivity in patients who had dystonia responsive to sensory tricks (“trick”) compared to those who did not (“no-trick”): in “no-trick” patients, there was increased functional connectivity between cortical domains (Figure 2A, top), while in “trick” patients there was reduced connectivity between sensorimotor and other (visual/executive) cortical domains (Figure 2A, bottom left). Moreover, the connectivity was further reduced when the “trick” patients used their effective gesture (Figure 2A, bottom right) [93]. In regards to the broader dystonia network, the authors made the interesting observation that while “no-trick” patients had increased connectivity between the cerebellum and motor cortex compared to “trick” patients, the sensory trick itself led to increased cerebellar activity (Figure 2A, bottom left) [93]. Consistent with these two studies, Murase et al. found that central gating of sensory cortical responses while preparing for movement was abnormal in patients with writer’s cramp, even when performing unrestricted, non-dystonic movements [98]. Finally, Gomez-Wong et al. found that in patients with blepharospasm there were abnormalities in the sensory portion of the trigeminal reflex arc that were more frequent in those patients who did not have an effective sensory trick [99]. The described studies highlight the complex interaction between brain regions during dystonia pathogenesis, implicate sensory and integrative cortical regions -in addition to motor cortex-in dystonia pathogenesis, and add further intrigue to the question of whether and how different forms of idiopathic and acquired dystonia relate to each other in onset, evolution, and response to treatment.

### Botulinum neurotoxin

Injection of botulinum neurotoxin (BoNT) into dystonic muscle groups is one of the key pharmacologic interventions for dystonia, especially for focal and segmental acquired dystonias [8]. BoNT was first used to treat blepharospasm in 1981 by Joseph Jankovic, who also published the first randomized controlled trial for its use, which directly preceded its approval in the United States for use in the treatment of dystonia [100]. The accepted, and primary mechanism of action for BoNT is via blockade of peripheral cholinergic neurotransmission [100]. This blockade is accomplished by interfering with the exocytosis of acetylcholine containing synaptic vesicles at axon terminal via proteolytic cleavage of SNAP/SNARE family proteins [100]. In the muscle, this mechanism of action prevents neurotransmission across the neuromuscular junction and leads to dose dependent muscle weakness. BoNT is a large protein complex that does not cross the blood brain barrier and thus does not directly target cholinergic neurotransmission in the central nervous system [100]. There have been numerous studies suggesting retrograde transport of BoNT from the site of injection via peripheral nerves, which would be an alternative route for a centrally acting mechanism of action [101, 102]. However, retrograde transport remains controversial, with other groups failing to note this phenomenon in muscle [103], or asking whether retrograde transport has clinically meaningful effects at therapeutic dosing in patients [104].

An alternative proposed mechanism of action posits that “deafferentation” via primary effects on gamma motor neurons and resulting secondary effect on the muscle spindle is responsible for altering sensory processing peripherally [105]. Regardless of the mechanism of action, including an entirely peripheral one, it is clear that long term central adaptation in the dystonia network occurs in association with BoNT [106]. However, there has been a great deal of difficulty disentangling directionality of network changes, which may point to differences within and among certain dystonia presentations (task specific focal dystonia [107, 108], blepharospasm [109], cervical dystonia [110]), timing of injection (BoNT treatment naïve [111, 112], non-naïve but before and after administration of BoNT [107, 110, 113], partial responders and responders [114]) or modality used to assay central network effects (resting state fMRI [65, 95, 111] vs. task dependent fMRI [107, 109, 110, 113], PET [108, 114], etc) [106].

We will summarize two of the most recent analyses regarding central effects of BoNT that each considered many of the above-described variables. The first, by Hok et al. [94], focuses on cervical dystonia, which has been linked both to disrupted cerebellar function and acquired etiologies. The second, by O’Flynn et al. [115], focuses on a task specific focal dystonia, whose underpinning etiology and associated networks have remained more enigmatic.

In their recent study examining idiopathic cervical dystonia, Hok et al. used resting state fcMRI to understand how BoNT administration altered central network function in those who responded to the therapy as compared to those who did not [94]. The authors chose the cerebellum as region of interest for the study based on previous reports implicating cerebellar function in cervical dystonia both in fMRI as well as *post mortem* histological studies [15, 116]. They found that in patients with a successful clinical response to BoNT, therapy led to decreased functional connectivity between the cerebellum and cortical areas in direct proportion to the efficacy of the treatment (Figure 2B, top) [94]. The authors also noted alterations in *intra*-cerebellar functional connectivity, in particular between regions that were previously associated primarily with motor function (right lobule VI) and those associated with cognitive function (right crus II) (Figure 2B, bottom) [94, 117]. In demonstrating tightly coupled changes in brain connectivity in response to BoNT administration in a network (cerebello-cortical) that has been associated with a particular type of dystonia (cervical dystonia), Hok et al. present a proof of concept of targeted inquiry that may yield fruitful insight into how to address the complexity of the etiological heterogeneity of dystonia presentations. Furthermore the finding of intracerebellar changes in functional connectivity is an interesting observation that aligns with a recent case report that investigated the efficacy of cerebellar *cortical* stimulation as a treatment for dystonia [118].

Another recent study, performed by O’Flynn et al., furthers this conceptual framework by tackling a similar question in laryngeal dystonia [115]. In this study, the functional connectivity of 161 patients with laryngeal dystonia who had received short, intermediate, or long-term treatment with BoNT were examined using fcMRI. They found that a cortical area (left precuneus) was an important brain region in laryngeal dystonia pathology and showed alterations in activity in response to BoNT in patients who had clinical benefit from the treatment [115]. O’Flynn et al. further noted that there were alterations in BoNT responders who had received therapy for an intermediate duration (6–12 years) in the cerebellum, including in lobule VI as noted by Hok et al. [94, 115]. Together these studies, which tackle etiologically and phenotypically distinct forms of dystonia, demonstrate a converging network of brain regions whose modulation seems to be critical in dystonia pathogenesis and perhaps also response to treatment.

Though the mechanism(s) for the central effects for BoNT in dystonia remains enigmatic, direct modulation of brain networks through DBS of target sites in the central nervous system is a key therapeutic intervention in refractory dystonias and offers direct evidence for how modulation of the dystonia network leads to amelioration of the movement disorder.

### Deep brain stimulation

DBS is an invasive neuromodulatory procedure involving the precisely targeted insertion of electrodes into target regions of the brain to deliver electrical current. DBS has become commonplace in the treatment of several movement disorders to include Parkinson’s disease, essential tremor, Tourette syndrome, and dystonia. Furthermore, DBS is increasingly being used in a wide range of other movement, cognitive, pain, epilepsy, and affective neurologic disorders [119–123]. Though a deep mechanistic understanding of how DBS achieves its therapeutic benefit remains elusive, the prevailing theory of its mechanism of action involves the disruption of the targeted nodes (“functional lesion”), while excitation and inhibition of the targeted and adjacent circuits may also be a key, region specific mechanism [119].

In dystonia, DBS (pallidal DBS in particular) is now a critical therapeutic intervention and is a first line consideration in certain generalized and segmental dystonia such as in the case of DYT*-TOR1A*, DYT*-KMT2B* and DYT-*SGCE* (Supplementary Video S4) [124, 125]. Though there is some debate on the issue, DBS is generally considered to be more efficacious in isolated (previously primary) dystonia as compared to secondary or acquired dystonias [126]. The most frequently targeted and highest efficacy brain region for dystonia treatment is the globus pallidus interna (GPi) although recent studies reveal equal efficacy and safety in subthalamic nucleus (STN) targeting [7, 127]. The benefit of GPi DBS on genetic dystonias is high enough that some investigators posit that genetic testing ought to be undertaken prior to all DBS implantation for prognostication of efficacy [126].

Meta analysis has shown that there is a durable long-term benefit to DBS in pediatric patients while also pointing out the relative poor efficacy of DBS in acquired dystonia as compared to the isolated dystonias [128]. With short term follow up, across studies using a common dystonia rating scale (the Burke-Fahn-Marsden Dystonia Rating Scale (BFMDRS)), inherited dystonias (DYT) showed ~40%–90% improvement in the BFMDRS motor scores with GPi DBS [128]; acquired dystonia had a lower, but still significant, response rate with BFMDRS motor score improvements of ~10%–30% [128]; and idiopathic dystonia showed an intermediate response of ~30%–70% [128]. With long term follow up, there was more heterogeneity within the DYT dystonias and the response to GPi/STN DBS was the highest in idiopathic dystonias (mean of 93%), followed by DYT-*SGCE* and DYT*-TOR1A* dystonia (mean of 89.2% and 80.7%), and finally acquired/CP dystonia (52.9%) [128]. Other inherited monogenic dystonias such as DYT-*THAP1*, DYT-*ATP1A3* and neurodegenerative dystonias (PKAN and Lesch Nyhan) had lower mean responses [128, 129]. Even though primary dystonia and dystonia resulting from acquired injury (CP) patients show reduction in BFMDRS score as a result of globus pallidus DBS, the acquired injury group failed to show concomitant improvement in dystonia related disability. The difference between BFMDRS score and related disability as well as differences within responses amongst monogenic dystonia highlights potential differences in underlying network dysfunction [130].

Given the high efficacy of pallidal DBS for idiopathic dystonia and the previous literature covering DBS in monogenic dystonias [131, 132], we will undertake a more focused view of the use of DBS in acquired dystonia, specifically in CP. Previously, several meta-analyses have evaluated the efficacy of DBS, generally targeting the GPi, for treatment of dystonia in CP [133, 134], which confirmed the above noted relatively lower efficacy of GPi in CP compared to primary dystonia. In addition to lower efficacy, others have noted that efficacy of DBS in dystonia may be more difficult to assess given the more protracted response to stimulation and the more subtle improvement than that noted in diseases such as Parkinson’s, where DBS can seem to work like a switch [135]. Furthermore, multiple groups have observed that GPi DBS for acquired dystonia seems to have not only lower motor efficacy when compared to other etiologies of dystonia, but it also fails to improve associated disability [130, 136]. Interestingly, recent studies have intimated the opposite effect with thalamic stimulation, whereby objective improvement of dystonia may be more limited but with greater improvement in dystonia associated disability [137]. However, other case series have noted that there is limited utility to subsequent targeting of the thalamus after failure of response to pallidal DBS [138]. Together these data raise a heretofore unmentioned aspect of dystonia: even in “pure” dystonias there are often co-morbid non-motor effects that ought to be considered when evaluating treatment efficacy; how this relates to dysfunction of the dystonia network across different etiologies, warrants ongoing, deep consideration.

Given the difficulty in treatment of acquired dystonia with either GPi, subthalamic, thalamic, or combinatorial DBS approaches, alternative sites of stimulation have been considered, fore among them nodes in the cerebellar network [139, 140]. Due to the first line therapies and DBS targeting being well established, cases with sole targeting of the cerebellum with DBS for dystonia treatment are difficult to find, but several case reports have noted improvement of severe dystonia with superior cerebellar peduncle and cerebellar nuclear stimulation after failure of GPi DBS [5, 141, 142]. Two of these reports contain interesting features that warrant further discussion. Horisawa et al. describe a case with sudden onset and rapid progression to severe symptoms, which is increasingly thought to be more descriptive of a functional/psychogenic etiology [141]. On the other hand, Lin et al. describe improvements in laryngeal and axial dystonia in the patient as well as improvement in spasticity, none of which respond well to DBS in general and DBS targeting GPi, STN, or thalamus specifically [142]. Combined, these unique effects of cerebellar DBS point towards the need for further investigation. While these two targets involve the cerebellar outflow tracts, an additional case series examined eight patients with spasticity where the cerebellar cortex (anterior lobe) was targeted and in addition to improvement in spasticity, improvement in co-existing dystonia was noted [143]. The variable response to DBS in acquired dystonias is not surprising given the previously noted observations regarding consistent involvement of components of the dystonia network (cortex, striatum, thalamus, cerebellum) across etiologies but varying response to treatment and the involvement of different regions. But this variability in response does beg for a more precise methodology for primary site targeting in acquired dystonia, such as was proposed previously with genetic testing in isolated dystonias prior to GPi DBS [126].

In terms of DBS mechanism of action, or at least relating to its effects on brain networks, the focus will turn towards two published studies examining how activity in the dystonia network was altered with use of DBS. In the first study, 15 patients with cervical dystonia and clinical response to DBS were evaluated using fMRI [96]. Scans were taken when “optimal settings” were activated and this was compared to “non-optimal settings,” and “DBS off” [96]. The group reported that optimal settings predominantly led to decreased activity in the sensorimotor cortex and their data appears to suggest that cerebellar activity is also consistently altered (predominantly increased; Figure 2C) with optimal settings compared to non-optimal or DBS off (from Figure 1 of [96], Panel D, left column). A second study published in the same year looked at 18 patients with dystonia of heterogenous etiology and evaluated both local and global functional connectivity as compared to control patients using fMRI [97]. This group found that DBS-ON compared to DBS off led to global (inter-) connectivity of subcortical networks approaching the activity patterns of healthy controls (Figure 2C) [97]. On the other hand, local connectivity within (intra-) both subcortical and cortical regions *diverged*, or moved further away in direction, from the activity in healthy controls, though the authors noted that if the data were stratified by clinical response, local connectivity patterns in those responding better to DBS did in fact approach patterns seen in healthy controls [97].

As genetic testing may offer prognostication about efficacy in the hereditary dystonias, so one can imagine clinically directed fMRI being a useful pre-surgical evaluation in acquired dystonias; while the groundwork for such a heuristic has been laid, more research in understanding interindividual differences and how networks respond to DBS will be necessary. Furthermore, as there is often significant functional impairment in these patients, acquisition of fMRI data would be complicated by possible requirement of adjunctive sedatives during imaging and absence of task dependent fMRI data.

Together the summarized perspectives from human studies of acquired and idiopathic dystonia reveal the emergence of a dystonia network that is involved across etiologies of the disease. Furthermore, whether the etiology of the dystonia emerges from neonatal injury, focal lesions in later life, or functional aberrations in networks--and whether the alleviation of symptoms results from the geste antagoniste, botulinum neurotoxin, or DBS--the brain networks involved in dystonia seem to converge on a shared dystonia network involving the striatum, thalamus, and cerebellum and the various cortical networks involved in sensorimotor processing and integration. Next, a more fine-grained examination of these network manipulations will be taken through investigation of the literature looking at network and lesion-based dystonia models in rodents.

## Rodent models of functional and acquired dystonia

Current animal models of dystonia aim to 1) model the functional network disruptions arising from the various etiologies of dystonia that are seen in humans or 2) test whether and how experimental manipulation of the networks implicated in dystonia produce the movement disorder. However, understanding how broad, non-specific alterations in a network lead to dystonia becomes difficult in the case of task dependent dystonia, genetic dystonia (where the implicated gene is often widely expressed in the brain), and dystonia resulting from non-focal lesions (as in the case of dystonic CP). As a result, the use of focal, inducible lesions in mouse models is an especially useful emerging approach to begin answering how focal network disruptions lead to dystonia.

**FIGURE 3:**
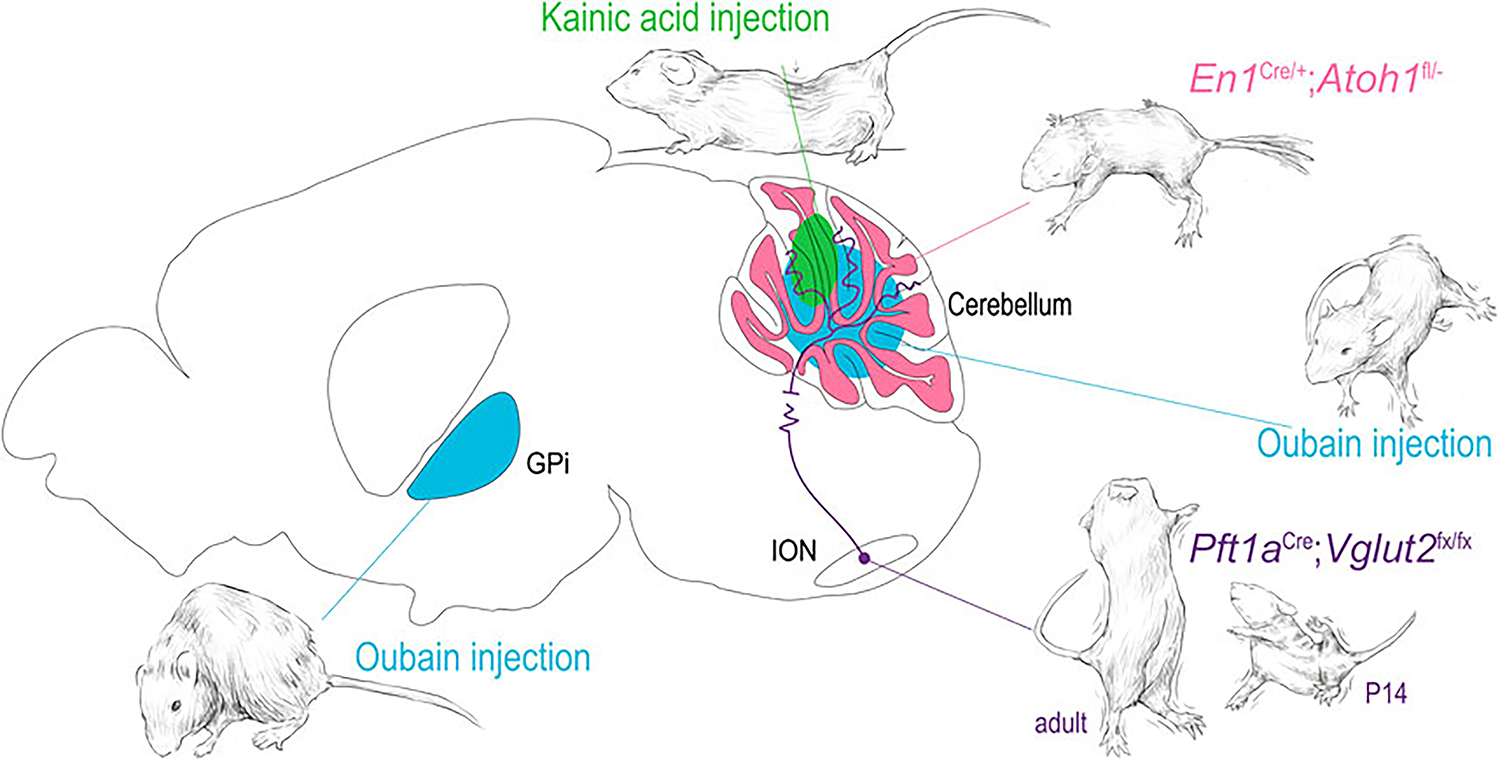
Dystonia arising from focal manipulations in the mouse. Using examples from the indicated publications, examples of dystonia are shown arising from 1) oubain injection (blue) in the basal ganglia and cerebellar vermis [144, 145]. 2) kainic acid injection into the cerebellar vermis [146] 3) functional disruption of the olivary-cerebellar projections [147] and 4) disruption of cerebellar granule cell development [148]. The drawings in this figure are artistic interpretations of the dystonic posturing of the mice as published in [144, 145, 149, 150], they were made in Procreate Version 5.3.5. The figure was compiled in Adobe Illustrator Version 24.0.1.

Mouse models of non-mendelian acquired dystonia often use paradigms involving pharmacologic network manipulation. Two of the most common pharmacological dystonia-inducing agents are the excitatory glutamate agonist kainite and selective sodium channel blocker ouabain, which have been shown to induce a range of dystonic phenotypes in mice (Figure 3) [61, 144, 145]. In the study conducted by Calderon et al., ouabain was noted to elicit dystonia from the selective blockade sodium channels in Purkinje cells, directly linking cerebellar dysfunction and dystonia. Pizoli et al. infused kainite into the cerebellar vermis and found subsequent dystonic posturing (Figure 3). Given the complexity of the cerebellar circuit and the gross imaging modalities used to link cerebellar function to dystonia in clinical studies, it is difficult to parse how these experimental manipulations correlate directly to the activity changes seen in patients, but it is a proof of principle for the utility of using focal manipulations of the dystonia network to test whether disruptions of nodes in the circuit can produce dystonia.

The use of pharmacological manipulations in mice has several distinct advantages. The selective induction of dystonia by blocking specific chemical receptors or pathways has allowed researchers to uncover several promising mechanistic pathways that could prove useful in the development of treatments for dystonia. For example, the exploration of the selective GABAergic transport inhibitor drug tiagabine was first tested in adult rats with a kainic acid-induced dystonia [146] and dopamine receptor agonist treatments were tested in mice in a kainic acid paradigm meant to model human idiopathic dystonias (e.g., blepharospasm, cervical dystonia, and spasmodic dysphonia) [151]. More specifically, Wang et al. [146] demonstrated that intraperitoneal injections of tiagabine into rats with kainic acid-induced lesions of the cerebellum improved locomotor function as measured in a beam walking task. Implicating the striatum in mouse models of dystonia, Fan et al. [151] showed that enhancing striatal dopamine neurotransmission could reduce the severity of induced dystonias as assessed by researchers in a blinded observation task.

Perhaps more importantly, pharmacologically induced dystonia models have been used to target specific anatomical structures in their investigation into dystonia. Using pharmacological lesions, rodent work has managed to elucidate key signaling biomarkers and mechanistic hallmarks of various dystonias in the cerebellum and basal ganglia [20, 152, 153]. Neychev et al. first highlighted the cerebellar-basal ganglia circuit as being integral to the production of dystonic movements as cerebellum-originating dystonia was exacerbated by subclinical striatal lesions and alleviated by cerebellectomy [20]. Fremont et al. identified that in mice with ouabain-induced lesions the presence of dystonia was accompanied by persistent high-frequency bursts of cerebellar nuclear neurons, and restoration of the ouabain-blocked sodium channels alleviated the symptoms of dystonia as assessed with rotarod and observation assays [152]. Georgescu Margarint et al. created an electromyographical setup that showed lateralized or vermal cerebellar dysfunction (due to kainic acid administration) ultimately triggered dystonia that was associated with a loss of connectivity in the corresponding cortical motor cortices (Figure 3) [153, 154], mirroring the human imaging studies in dystonic CP and task specific dystonias. Such studies have highlighted the evolutionary conservation of the dystonia network and shown that anatomic and circuit derived manipulations of the network can inform and confirm findings in the clinical population.

As the study of lesion-acquired dystonia in mouse models continues, there are several key factors to keep in mind. Researchers attempting to classify irregular motor behavior or changes in locomotion as the result of lesion-acquired dystonia should be as specific as possible when assessing 1) what manipulations were used; 2) how the manipulation might impact multiple areas of the brain with particular attention to the network level changes involved; and 3) what the assays they are using to evaluate symptoms of dystonia are truly assessing. For example, the use of pharmacological lesions to induce dystonia may not necessarily be the most representative method when modeling dystonias associated with traumatic brain injury (TBI) or damage resulting from central nervous system pathologies such as stroke, multiple sclerosis, or cerebral palsy. In these cases, a viable alternative in mouse models may be direct electrical stimulation to disrupt normal signaling processes as described in Raike et al., where a combination of conditional genetic and electrical stimulation was used to produce dystonia in proportion to the amount cerebellar dysfunction induced [155]. Another consideration is the quantification/evaluation of the dystonia produced by the manipulation, which usually comes in the form of the dystonia rating scale or various motor performance assays. With a dystonia rating scale, as described previously [145], the degrees of severity and types of dystonia can be standardized across observations. While this qualitative system is subject to the researcher’s discretionary bias or interpretation, actions such as blinded observation tests or collecting visual examples of the dystonia rating scores as they are given for future review can be implemented as well. For symptomatic mouse models of dystonia, researchers evaluating motor deficits of generalized dystonia often use multiple assays that target different aspects of motor function when classifying a phenotype as “dystonia”. Examples of individual assays that can be combined include pole climbing, rotarod, and open field assays as described in Fernagut et al. [156], gait analysis using a treadmill or foot-printing assay as described in Calderon et al. [144] or Aissa et al. [157], a “skilled reaching” task as described in Kernodel et al. [158], EMG and tremor recordings as described in Brown et al. [149], and developmental motor reflexes as described in Van der Heijden et al. [159].

### Dystonia network manipulations in mice

In addition to the work mentioned above, which focused on understanding the impact of manipulations of key cell types, brain regions, and pathways in the broader dystonia network, work in mouse models focusing on precise manipulations of the structures and circuits associated with dystonia have allowed us to better understand how dysfunctions within a broad dystonia network may develop. White and Sillitoe focused on investigating how functional disruption of projections from the inferior olive to the cerebellum contribute to dystonia (Figure 3) [147]. Using a transgenic mouse model that allowed for targeted silencing of the excitatory projections from the inferior olive to the cerebellum, they showed that precise manipulation of a single neural pathway was sufficient to produce dystonia [147]. Using *in vivo* electrophysiology, the authors found that dystonia-like behavior was mediated by abnormal firing activity in cerebellar Purkinje cells of juvenile mice and notably persistent abnormal activity of cerebellar nuclear neurons in adult mice. These findings confirmed prior observations in the spontaneous dystonic rat, *dt*, which also displays loss of climbing fiber activity, and subsequent abnormal Purkinje cell and cerebellar nuclei neuron firing activity [150, 160].

Further investigation of cerebellar circuitry by Van der Heijden et al. [148] found, using an alternative targeted genetic manipulation, that the disruption of granule cell neurogenesis led to altered Purkinje cells firing through an alteration of their developmental timeline (Figure 3). These changes together resulted in broader deficits in cerebellar function and behavior, including dystonic postures. This model, with altered cerebellar cortical function and resulting pervasive behavioral phenotype including dystonia, may inform our understanding of dystonic CP by modeling the developmental disruption though to occur through the various acquired insults to which the cerebellum is susceptible in early life.

The intersectional genetic models that cause functional lesions can further provide insight into the etiologies of symptoms that are often comorbid with dystonia [161]. For example, by studying the White and Sillitoe genetic dystonia model, Salazar-Leon and Sillitoe found these dystonic mice also exhibit sleep disturbances [162]. Interestingly, these sleep disturbances are also observed in a model where the genetic manipulation affects fewer cerebellar inputs and mice do not display overt dystonic features. This suggests that cerebellar dysfunction, and not the motor disturbances exclusively, are the main driver of comorbid sleep deficits in these mice. Similar studies can be performed to disentangle the neural underpinnings of other comorbidities often observed with dystonia, including mood disturbances and pain [162].

Again, using the targeted genetic approaches available in rodent models, a synthetic approach to model and treat “acquired” etiologies of dystonia is here given a proof of principle. These data may further add weight to diversifying the targets for interventions in the difficult to treat realm of acquired dystonias. Continuing to focus on 1) how the combinations of various anatomical changes to the structures underlying a broad dystonia network result in the different features of various dystonias and 2) how these translated behaviors can be abolished or mediated by therapeutic responses could reveal the causative mechanisms and or risk factors in human dystonias and their related conditions.

## Discussion

Recent efforts to characterize the dystonia network based on etiology, phenomenology, therapeutic intervention, and animal modelling have yielded a great deal of insight into the nodes that comprise the dystonia network and how they respond to insult and intervention. The most severe of the described dystonias, dystonic CP, often involves simultaneous pathology to multiple structures of the dystonia network due to the susceptibility of the immature brain to the various environmental insults present during neonatal life. By contrast, focal lesions into adulthood have a more predictable phenomenological presentation as described by Corp et al. and depicted in Figure 1, [41]. An interesting observation made by Corp et al., is regarding the varied latency to dystonia onset by lesion location; understanding why certain lesions, depending both on the type of insult (ischemic, hemorrhagic, inflammatory, etc.) and location, produce dystonia on different time scales may help to parse the functional roles of the individual node in the genesis of dystonia and what role the adaptation to brain injury has on the genesis of dystonia. Finally, isolated dystonia, whether idiopathic or task dependent, offers a unique perspective on the acquired dystonias as isolated dystonia is provoked by induced aberrant network activity rather than structural network disruption. Even in cases where there is an underlying genetic abnormality, the incomplete penetrance of the phenotype combined with the emergence of dystonia with task overuse offers an interesting flashpoint in understanding how the nodes in the dystonia network interact to produce dystonia (both structurally and functionally).

Functional imaging of therapeutic interventions has offered considerable insight into how the dystonia network can be modulated to alleviate the symptoms of the disease. A consistent thread across modalities is altered cerebellar activity, with both effective sensory trick and efficacious DBS resulting in increased cerebellar activity. In addition, decreased functional connectivity between the cerebellum and the cortex is seen in both successful BoNT therapy as well as in patients who have dystonia responsive to sensory tricks. Together, the anatomic and functional findings made when studying varying etiologies and treatment modalities both confirm the anatomic and functional substrates of dystonia and make it increasingly clear that more work needs to be done in understanding the precise role of each of the nodes in generating the dystonic phenotype; for instance how do the observations of increased cerebellar activity in response to therapy and the decreased functional connectivity between the cerebellum and cortex relate to one another? Can the cerebellum play a role in plastic network function, being involved in both the genesis and resolution of the movement disorder? Even with the wide and increasing breadth of literature covering dystonia, more work needs to be done to understand how these crucial brain areas work to buttress each other in both health and disease.

Rodent models are perhaps an ideal starting point for these investigations. As described, rodent models have the unique benefit of allowing targeted network manipulations in the mammalian nervous system. The genetic toolkit available in murine genetics has revealed dystonia arising from the functional manipulation of projections from single populations of neurons, which should serve as a proof of principle for ongoing studies in understanding the necessity and sufficiency different nodes in the dystonia network to produce the disorder. Furthermore, combinatorial pharmacologic neuromodulation, as described above, has made inroads towards understanding how different nodes can modulate one another.

## Concluding thoughts

From the heart-rending sight of a child in the midst of a dystonic crisis to the loss of livelihood suffered by a vocalist with laryngeal dystonia, the impact of this enigmatic and unfortunately prevalent disorder can be difficult to overstate. Dystonia can be a co-morbid movement disorder that compounds and exacerbates the complex needs of children with severe neurodevelopmental disability; it can manifest as a provoked attack in children with CP, causing anxiety and robbing joy from moments of celebration; for adults it can disrupt basic activities of life: blepharospasm causing one’s loss of independence through an inability to drive; task specific focal dystonia ending a promising career. However, progress towards understanding the network basis of dystonia is being made through the concerted effort of an expanding field of researchers. The increasing consensus implicating dysfunction in a broad dystonia network across many etiologies of this disorder should help to sculpt therapeutic modalities toward targeted interventions.

Furthermore, while mentioned only briefly in the review, there is an increasing awareness that dystonia, though presented and conceptualized as a movement disorder, may involve many non-motor domains and lead to emotional, autonomic, cognitive, and sleep disorders [163, 164]. While this may be a surprising observation in the framework of previous conceptualizations of dystonia, the perspectives and findings described in this review, in which widespread changes in broad brain networks are manifest, perhaps shed light on why patients suffering from dystonia may display these symptoms. Even if we may increasingly think of dystonia as a disorder of sensory motor integration, perhaps it may be even more apt to think about dystonia as a disorder of network integration, with disrupted coordination of networks manifesting most obviously, phenomenologically, in the movement disorder of dystonia but more subtlety across nearly all other functional domains.

## Supplementary Material

Video 1 **SUPPLEMENTARY VIDEO S1** Generalized dystonia in dystonic/dyskinetic cerebral palsy: Apparent is severe axiadystonia with opisthotonic posturing, retrocollis, torticollis, and oromandibular dystonia. Also present are stereotypies of the upper extremities.

Video 2 **SUPPLEMENTARY VIDEO S2** Hemidystonia due to tuberculous meningitis: This patient has left sided appendicular dystonia resulting from an acquired brain injury. Evident is dystonic posturing of the left hand, arm, leg, and foot. Dystonia worsens with action, in particular walking, while walking backwards there is slight improvement in gait.

Video 3 **SUPPLEMENTARY VIDEO S3** Functional focal dystonia. This patient has focal, fixed, right foot inversion and inward rotation of at the hip with acute onset. There is no fluctuation of the dystonia and when the patient turns with walking the foot returns to normal position briefly and then the fixed position. The findings are consistent with a pure functional dystonia.

Video 4 **SUPPLEMENTARY VIDEO S4** Generalized *Tor1A* dystonia responsive to GPi DBS. Pre-DBS video shows a generalized dystonia with severe restriction of movement with significant pain. Post-DBS the patient has achieved significant improvement in motor function as is evident through the comparative examination as well as the gait performance.

Video 5 **SUPPLEMENTARY VIDEO S5** Segmental *Thap1* dystonia. This genetic dystonia can cause focal/segmental dystonia which is evident in the patient’s cervical dystonia (laterocollis and torticollis), oromandibular dystonia, toe extension dystonia, and voice abnormality (laryngeal dystonia).

## Figures and Tables

**TABLE 1:** Examples of different dystonias based on Axis 1 and Axis 2 classification.

	Axis 1 (presentation)	Axis 2 (etiology)

Patient/Supplementary Video S1	Generalized	Cerebral Palsy
Patient/Supplementary Video S2	Segmental	Acquired Brain injury
Patient/Supplementary Video S3	Focal	Functional
Patient/Supplementary Video S4	Generalized	Genetic
Patient/Supplementary Video S5	Focal/Segmental	Genetic

Included are videos demonstrating dystonia phenotypes based on their phenomenology (indicated in the middle column) and their etiology (indicated in the right column). All videos were obtained by Dr. Mara Hull in the Movement Disorder Clinic at Texas Children’s Hospital and consent for inclusion in research studies was obtained from the patient and/or legal guardian.
